# EDGAR3.0: comparative genomics and phylogenomics on a scalable infrastructure

**DOI:** 10.1093/nar/gkab341

**Published:** 2021-05-14

**Authors:** Marius Alfred Dieckmann, Sebastian Beyvers, Rudel Christian Nkouamedjo-Fankep, Patrick Harald Georg Hanel, Lukas Jelonek, Jochen Blom, Alexander Goesmann

**Affiliations:** Bioinformatics & Systems Biology, Justus Liebig University Gießen, Heinrich-Buff-Ring 58, 35390 Gießen, Hesse, Germany; Bioinformatics & Systems Biology, Justus Liebig University Gießen, Heinrich-Buff-Ring 58, 35390 Gießen, Hesse, Germany; Bioinformatics & Systems Biology, Justus Liebig University Gießen, Heinrich-Buff-Ring 58, 35390 Gießen, Hesse, Germany; Institute of Computational Biology, Helmholtz Center Munich, German Research Center for Environmental Health, Ingolstädter Landstraße 1, D-85764 Neuherberg, Germany; Bioinformatics & Systems Biology, Justus Liebig University Gießen, Heinrich-Buff-Ring 58, 35390 Gießen, Hesse, Germany; Bioinformatics & Systems Biology, Justus Liebig University Gießen, Heinrich-Buff-Ring 58, 35390 Gießen, Hesse, Germany; Bioinformatics & Systems Biology, Justus Liebig University Gießen, Heinrich-Buff-Ring 58, 35390 Gießen, Hesse, Germany

## Abstract

The EDGAR platform, a web server providing databases of precomputed orthology data for thousands of microbial genomes, is one of the most established tools in the field of comparative genomics and phylogenomics. Based on precomputed gene alignments, EDGAR allows quick identification of the differential gene content, i.e. the pan genome, the core genome, or singleton genes. Furthermore, EDGAR features a wide range of analyses and visualizations like Venn diagrams, synteny plots, phylogenetic trees, as well as Amino Acid Identity (AAI) and Average Nucleotide Identity (ANI) matrices. During the last few years, the average number of genomes analyzed in an EDGAR project increased by two orders of magnitude. To handle this massive increase, a completely new technical backend infrastructure for the EDGAR platform was designed and launched as EDGAR3.0. For the calculation of new EDGAR3.0 projects, we are now using a scalable Kubernetes cluster running in a cloud environment. A new storage infrastructure was developed using a file-based high-performance storage backend which ensures timely data handling and efficient access. The new data backend guarantees a memory efficient calculation of orthologs, and parallelization has led to drastically reduced processing times. Based on the advanced technical infrastructure new analysis features could be implemented including POCP and FastANI genomes similarity indices, UpSet intersecting set visualization, and circular genome plots. Also the public database section of EDGAR was largely updated and now offers access to 24,317 genomes in 749 free-to-use projects. In summary, EDGAR 3.0 provides a new, scalable infrastructure for comprehensive microbial comparative gene content analysis. The web server is accessible at http://edgar3.computational.bio.

## Graphical Abstract

**Graphical Abstract:**
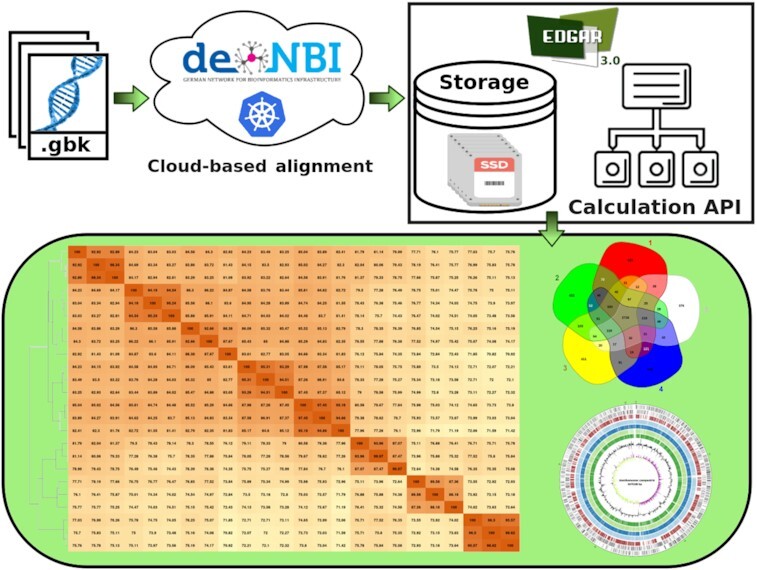
At the top a schematic overview of the cloud-based workflow and the new backend of EDGAR3.0 is shown. The bottom part of the figure shows a selection of EDGAR results for the genus *Xanthomonas*.

## INTRODUCTION

In the twelve years since its initial publication, the EDGAR platform has become one of the most popular services in the field of comparative genomics and phylogenomics. Ten years ago, the calculation of the genomic subsets pan genome, core genome and singleton genes (1) were the main feature of the EDGAR web server, and projects contained only 5–10 genomes on average. Over the last years, the focus of the EDGAR web server shifted more and more towards the field of phylogenomics, with whole genome or core-genome-based phylogenomic and taxonomic analysis becoming the main application field of the software. Today, the EDGAR platform offers a wide range of tools required for phylogenomic inter- and intraspecies taxonomic analyses, e.g, core-genome-based phylogenetic trees, average amino acid identity (AAI) and average nucleotide identity (ANI) matrices (2–4). The web server provides several visualization features such as multi genome synteny plots, comparative circular genome plots, or Venn diagrams to enable quick access to information about the differential gene content of kindred genomes and to gain insights into their evolutionary relationships.

Due to the rapid development and broad application of DNA sequencing technologies, the number of genomes that are processed on the EDGAR platform is steadily increasing. In 2009, the EDGAR platform comprised 75 projects with 582 genomes in total. As of March 2021, there are 1715 EDGAR projects, and the largest project alone for the genus *Escherichia* with 1326 genomes comprises more than twice as many genomes than the complete database of EDGAR1.0.

Naturally, this huge increase of dataset sizes poses a big challenge for the further development of the platform. On the one hand, EDGAR orthology estimation is based on an all-against-all BLASTP (5) comparison of amino acid sequences and requires substantial computational efforts.

The additional computational effort due to increasing sequence availability is partially compensated by the general development of computational capacities, but as the complexity of the alignment step is growing quadratically with the number of analyzed sequences, the computational requirements of the EDGAR platform have risen considerably over the last decade.

On the other hand, the storage capacity needed to store the results and the memory requirements for queries against the increasing amounts of data were rapidly growing. Since EDGAR version 2.0, the pre-calculated BLAST result were filtered and subsequently stored in a MySQL database. For projects with hundreds of genomes, the number of stored BLAST results can easily reach hundreds of millions. As a consequence, the MySQL backend became more and more of a limiting factor for the EDGAR web server as the data import and indexing are very time consuming efforts. Finally, the access to the underlying data of the EDGAR platform stored in a classical relational database model was becoming a critical bottleneck with regard to processing time as well as memory consumption for larger projects. All these issues can be expected to become even more problematic with the projected growth of genomics data in the future, which is even supposed to outgrow Moore’s Law (6). To overcome the described challenges, we developed a modernized, scalable backend that allows us to ensure the operation of the EDGAR web server and to provide the EDGAR service to the scientific community in the future.

## MATERIALS AND METHODS

The new EDGAR3.0 backend tackles the three main problems of the previous versions: The need of extensive compute resources for the intial alignment step, the demand for a performant data storage solution with quick, parallel data import, and the need for rapid and memory efficient processing of the different analyses of the EDGAR3.0 web server. The details of the EDGAR3.0 backend as well as new analysis features of the web server will be described in the following sections.

### BLAST calculation

Calculating the required BLAST hits between all genes of a project is a challenging task and needs significant computational resources. In the previous years, the number of available genome sequences increased drastically, and as a result the number of genomes (and subsequently genes) per EDGAR project grew accordingly. The number of aligments needed for the setup of an EDGAR project grows quadratically with increasing number of genes in a project, thus it was crucial to implement a method to run these calculations in a timely and efficient manner and in a way that is scalable with the number of input genomes. Consequently, a small tool has been developed that distributes the BLAST computations across an arbitrary number of cores in a Kubernetes cluster (https://kubernetes.io). Currently, a cluster with 3000 cores running in the de.NBI cloud is used, with the option to increase the number of cores if required. The Kubernetes BLAST tool places the sequence data of an EDGAR project in an S3 object storage. A BLAST index is created for the sequence data and stored in a shared file system that is accessible in the whole Kubernetes cluster. The data is then split into chunks, and each chunk is analyzed by a Kubernetes Job in the cluster in parallel. The BLAST binaries used in the Kubernetes implementation are identical to the ones used in the legacy implementation, thus results are also identical. The results are written back to the object storage. From there they are converted into the new storage layer format.

### New Storage backend

**Figure 1.**
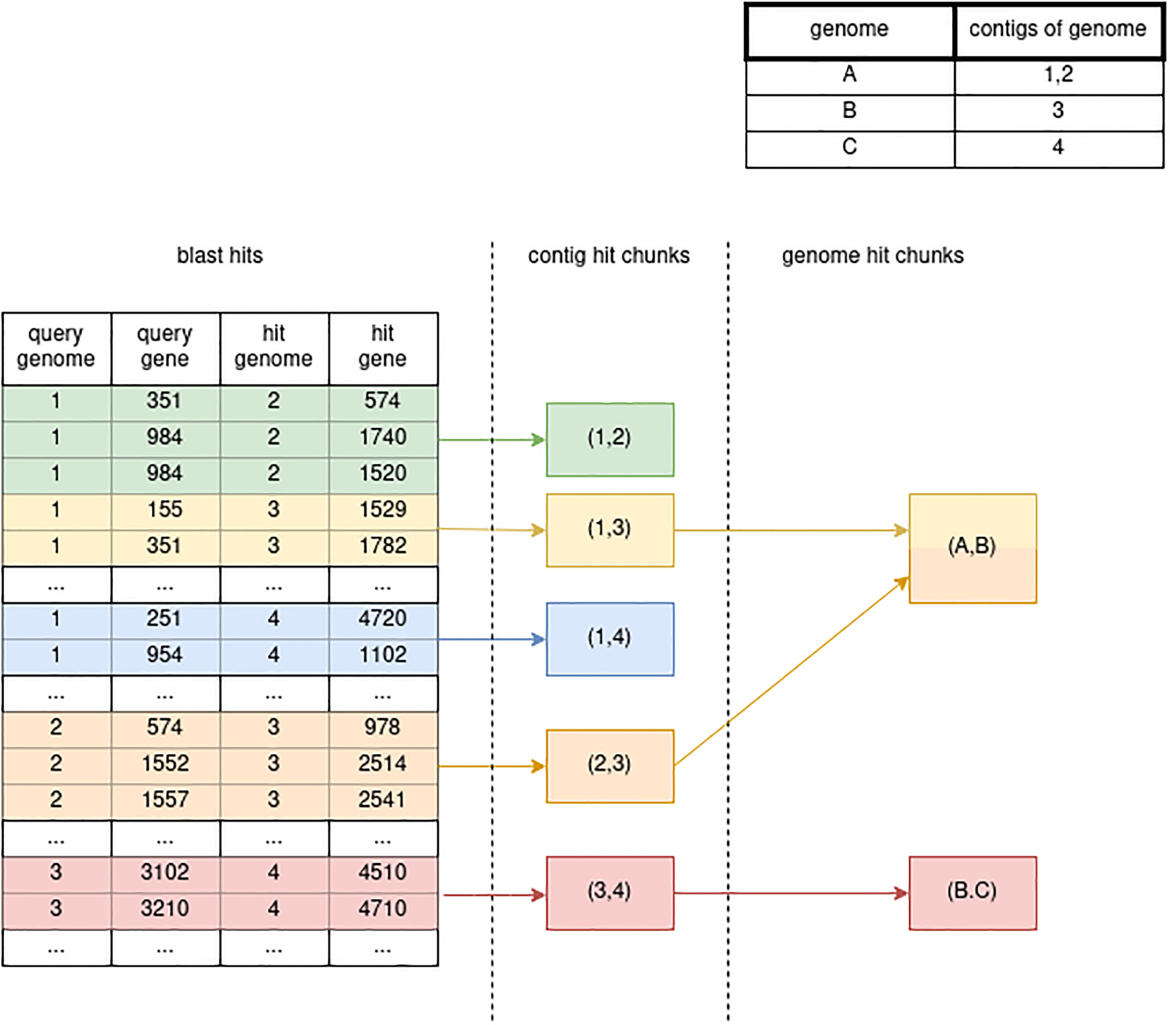
Grouping of blast hits in the storage backend and for genome grouping. Hits with the same query contig id and hit contig id are grouped together into contig hit chunks (colored areas). 1...*n* contig hit chunks can be combined to a genome hit chunk representing the hits of a genome against another genome. Genome A is a set of contigs {1,2}, Genome B of contig {3} and Genome C of contig {4}

The new storage backend for EDGAR3.0 has been implemented based on the fast and efficient data format protocol buffers. The new data backend relies on the grouping of individual BLAST results based on the contigs of origin of query and hit gene. All BLAST results are sorted according to a tuple of these IDs of the query contig and the hit contig and clustered into so called *contig hit chunks* accordingly. Subsequently, all contig hit chunks are stored in one single file (Figure 1, left part). The byte positions of the individual chunks are written to a separate index to allow quick access to individual hit chunks using two-tuple of query and hit id representing the contig hit chunk as keys. The index and data files are stored in a SolidFire All-Flash-Storage (https://www.netapp.com/de/data-storage/solidfire/) solution to ensure rapid access for all queries of the EDGAR3.0 web server.

#### Genome grouping

As a microbial organism might comprise more than one replicon, EDGAR allows for the analysis of the combination of all replicons of an organism. To facilitate this, the calculation of orthologs is performed on genomes rather than contigs. A genome is a set of 1...*n* contigs. If all contigs of an organism are part of a genome, it is called organism genome. For the calculation of orthologs the contig hit chunks are merged into genome hit chunks that contain all hits from all contigs of both genomes. For example the genome hit chunk of genome A in genome B, with A being a set of contigs {1, 2} and B of contig {3}, contains the following contig hit chunks: {(1, 3), (2, 3)}. The genome hit chunk of B in A contains {(3, 1), (3, 2)} (Figure 1, right part).

### New calculation backend

Based on this modernized storage backend, a novel ortholog calculation algorithm based on the divide and conquer principle has been implemented. The new implementation reduces the memory complexity and makes heavy use of parallelization to reduce calculation times of large computational tasks with a drastically lower memory consumption.

**Figure 2.**
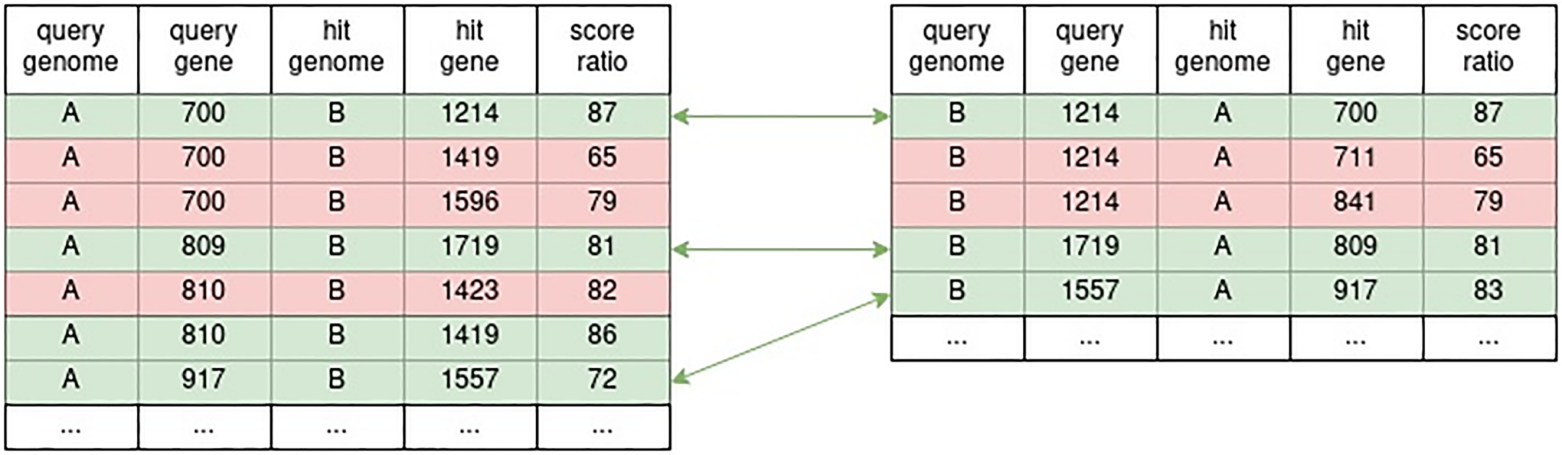
Ortholog calculation on a reciprocal tuple of genome hit chunks. At first the best hits of each query gene are selected (green hits) in both chunks. Then the best hits of both genome hit chunks are matched against each other. Those with reciprocal partners are accepted as ortholog hits (green arrows).

The ortholog calculation is based on reciprocal best blast hits as described in the orignal EDGAR paper (7). The new implementation computes orthologs on reciprocal two-tuples of the genome hit chunks described in the previous section. To calculate the ortholog of a given set of genomes, all combinations of these tuples have to be processed. After computing all reciprocal two-tuples the required contig hit chunks are read from the storage layer and merged into corresponding genome hit chunks. All further ortholog calculation is performed on these individual two-tuples in parallel and independently of others. The parallelism is tuned for optimal performance with regard to the current load and memory usage of the backend server and is automatically adjusted to changes in these metrics. Within the two-tuple two steps have to be performed in order to calculate orthologs. At first the best hit of each gene is selected within each genome hit chunk. For each best BLAST hit in the genome hit chunk its reciprocal counterpart is scanned for a reciprocal best BLAST hit. If a matching pair is found, the genes of the hit pair are accepted as orthologs (Figure 2). The new calculation backend was designed to be as compatible as possible to the algorithmic ideas and methods that were described in the original EDGAR paper (7) and subsequent updates (8,9). Nonetheless, additional gene comparison criteria were introduced to the algorithm, though, which helps to find the best hits more reliably in rare edge cases. Some calculations require the selection of a reference genome, which defines the starting gene set for the iterative calculation of, e.g. the core genome or pan genome.

### New backend API

To facilitate communication between the web server and the new backend and to allow the server to call the backend analysis functions, a JSON-over-HTTP based REST API is provided by the new backend. This API offers interfaces for the ortholog calculation and all higher level functions required by the EDGAR3.0 web server with its analysis features and visualizations. It also allows access to the data stored in the new storage backend. Functions offered by the API include the calculation of the core and pan genomes and singleton genes, the AAI and POCP calculation, core/pan/singleton development plots, the venn diagram data, and ortholog set calculations.

### New analysis features

The features of the EDGAR web server have been described in three previous publications (7–9), thus we will not describe all features in detail, but will focus on some of the recent additions to the EDGAR feature set.

#### Upset intersecting gene set plots

A method to get a visual representation of intersecting sets for more than five datasets is UpSet (10). EDGAR 3.0 offers static UpSet images showing the genome combinations sorted based on the gene number for the respective combination generated by UpsetR (11). Furthemore, an interactive UpSet visualization is generated in which data can be dynamically sorted, aggregated and arranged according to the needs of the user.

#### Genomic distance metrics

As an addition to the existing genomic distance features AAI and ANI, the fastANI (12) approach, a fast alignment-free computation of whole-genome similarity values, as well as the calculation of the percentage of conserved proteins (POCP) between two genomes as proposed by Qin *et al.* (13), have been added to EDGAR3.0 The data needed for the POCP calculation can be directly extracted from the new EDGAR ortholog calculation backend. In the POCP publication, a set of fixed orthology cutoffs is used, i.e. an evalue threshold of 1*e*^−5^, a minimum sequence identity >50%, and an alignment coverage of the query protein >50%. EDGAR3.0 implements this method in two different ways: One uses the ortholog criteria defined in the original POCP paper, the other uses the established EDGAR ortholog definition established in the orignal EDGAR paper (7).

#### Comparative circular genome plots

A further feature added since the publication of EDGAR2.0 is the interface to generate comparative multigenome circular plots. EDGAR uses the BioCircos tool (14) to visualize the orthology information of several genomes in one image.

#### PSOS integration

To provide on-the-fly annotation information for genes, the protein sequence observation service (PSOS - https://psos.computational.bio/) was integrated into EDGAR3.0. The tools include a homology search against UniprotKB/Swissprot (15), a search against the PFAM-A database (16), signalpeptide prediction (17), transmembrane helix prediction (18) and cellular localisation prediction (19).

### Languages and libraries

To make the implementation of the new backend for the calculation of orthologs as well as all features dependent on these orthologs fast and efficient, it was necessary to choose an appropriate technology stack. The new algorithm and storage implementation are written in the programming language Go (https://golang.org/), which offers fast and efficient multithreading and memory management, an efficient co-routine scheduler, efficient garbage collection, simplicity and availability of required third-party frameworks. As part of the new backend, the new storage system uses a file based approach to store its data. The hits are stored in protocol-buffer format (https://developers.google.com/protocol-buffers/) to allow fast access and snappy compression (http://google.github.io/snappy/) to enable fast decompression with limited processing overhead when reading the data from the storage.

## RESULTS

The new technical backend of EDGAR3.0 allows the processing of much larger EDGAR projects and facilitated the major update of the public EDGAR database section, which tripled the number of genomes in the freely accessible database. The new backend also allowed us to implement new features as extension of the EDGAR3.0 web server.

### A scalable backend for large-scale genome comparisons

The new backend improves multiple important features of the EDGAR3.0 web server.

First of all, the computationally most intensive step, the initial calculations of the BLASTP results, was implemented in a cloud native architecture. This allowed us to use the computing capacities of the de.NBI cloud hosted by the German Network for Bioinformatics Infrastructure for this step. It thereby also ensures the easy scalability of the EDGAR project setup.

To address the data storage and access time issues, a file based storage solution using protocol buffers is used. The alignment results are no longer stored in a relational database management system, but in a file based backend that is stored on an ultrafast all-flash storage to ensure optimal access times. This drastically accelerated the setup times of EDGAR3.0 project in comparison to previous versions, as no database import is necessary and the filtering of the BLAST results is considerably faster. While the largest project hosted on the old backend, a private project of *Streptococcus* genomes, consisted of 1 billion BLAST results, the largest project on the new backend, the public *Escherichia* project, comprises 13.1 billion BLAST results. At the same time, the setup of the Escherichia project took only half the time of the *Streptococcus* project.

Based on this fast storage backend, EDGAR3.0 can now calculate orthologs across any number of genomes with a constant memory complexity of $\mathcal {O}(1)$ with regard to the number of processed genomes. The new storage backend is optimized for a scalable and highly parallel execution of EDGAR queries. It reduces the calculation time to determine orthologs due to its ability to parallelize the computation across all cores available to the server and its use of a compiled programming language. On the old backend, the limit for pan genome calculation was between 150 and 200 genomes, depending on the genome size, before the computation became too lengthy to be finished in reasonable time for the web server. On the new calculation backend, the complete pan genome of 881 *Bacillus* genomes, a matrix of 69.6 million entries, can be calculated in about 50 minutes.

### New features of the web server

While the technical upgrades are in the focus of this work, there are also some new features that extend the functionality of EDGAR3.0. The addition of fastANI and POCP further strengthens the set of features for phylogenomics, the main application field of the EDGAR platform. POCP provides a further method to estimate the evolutionary and phenotypic distance of genomes. While the data needed for the POCP calculation can be directly extracted from the new, fast EDGAR backend, the fastANI results are computed on-the-fly for up to 200 genomes as soon as a user queries a fastANI matrix. POCP results as well as fastANI results are presented as heatmaps comparable to the ones already provided for AAI and ANI.

**Figure 3.**
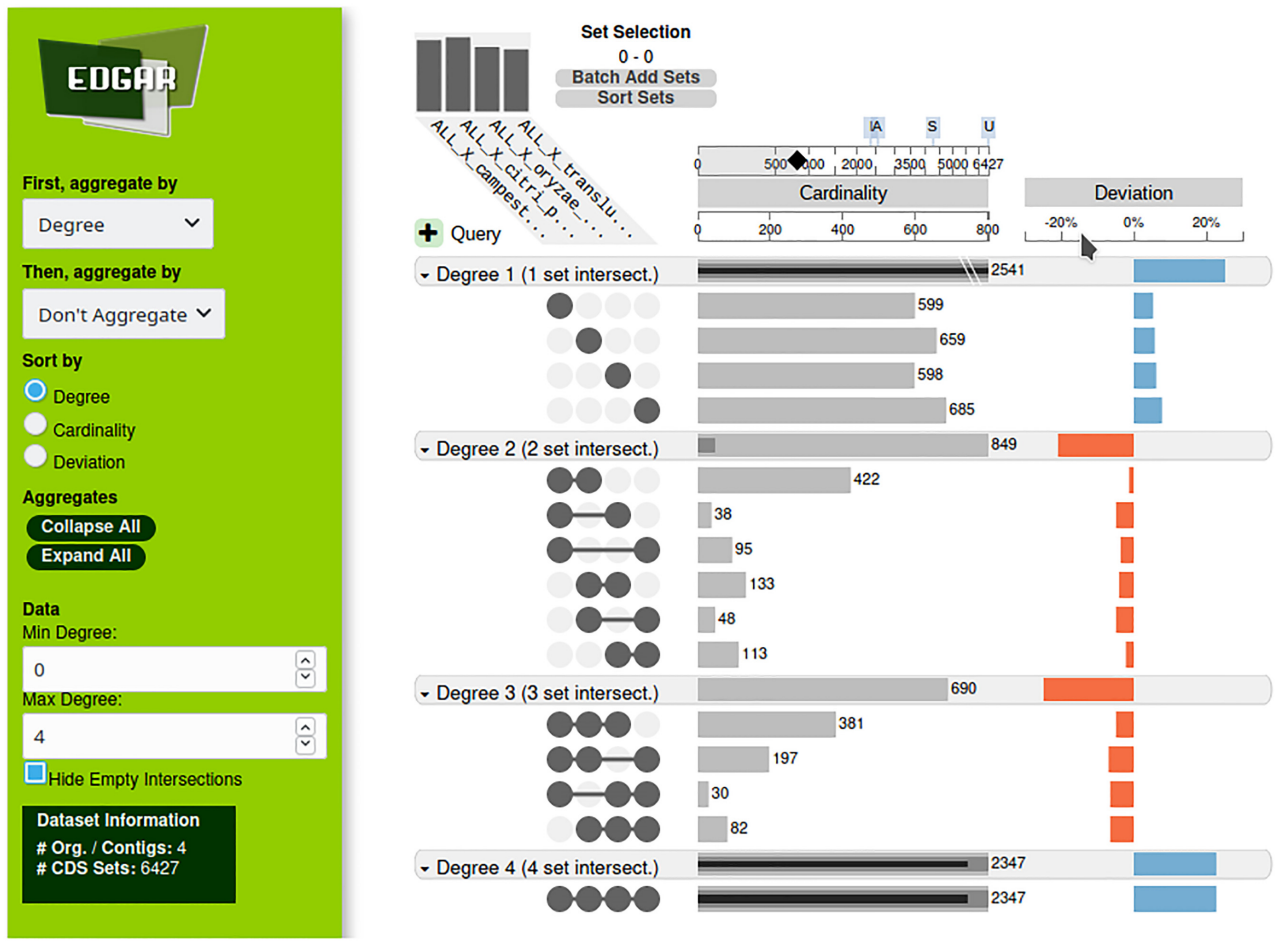
Upset intersecting gene set plot. Results for the intersecting genes of four *Xanthomonas* genomes are shown. In this fully interactive plot, results can be sorted according to degree, cardinality or deviation. Data can also be aggregated based on the respective attributes, and the bar plots can be scaled dynamically.

Classical methods to display the intersections of several datasets like Venn diagrams tend to get overloaded and fuzzy pretty quickly. Thus Venn diagrams of more than five sets are rarely seen—EDGAR also allows a maximum of five sets in its Venn diagram implementation. The new visualization features of the UpSet plots and the circular plots are designed to allow for the visual inspection of shared and differential gene content of larger genome sets. UpSet (10) is a method that generates a clear visual representation of intersecting sets for more than five datasets. In Upset, intersections are in a matrix layout where dark grey circles represent the genomes included in a set, while missing genomes are visualized as light grey circles (Figure 3). The UpSet visualization makes it easy to quickly get insights into the distribution of genes among a set of genomes.

Circular genome plots are used to visualize regions of interest on genomic sequences (20–22). This approach can also be used for comparative visualizations by combining several circular genome representations to highlight common features of the respective genomes. EDGAR provides multi genome circular plots to visualize the orthology information of several genomes in one image. The outer rings of the circular plot represent the genes of one selected reference genome. The further rings of the circular plot show the core genome as well as the orthologs of each individual genome in comparison to the reference. The circular plot is completely interactive and allows users to get information about every single gene in the plot via a hover box. It is also possible to zoom in and out to specific areas of the plot. The plots can be exported as SVG vector graphics.

During the project setup, EDGAR extracts the annotation of all coding sequences that are imported from the Genbank files provided by the user. Depending on the source of the genomic data, the annotation might be several years old at the time of the EDGAR import. Even if the annotation is up-to-date while the EDGAR project is created, new sequence data and new functional annotations of proteins become available every day. Thus, it is desirable to discover sequence similarities to novel entries in public databases such as Swissprot (15) or Genbank (23). Furthermore, we were looking for a way to assist users in the assignment of functions to genes that were so far annotated as *hypothetical proteins* or *proteins of unknown function—*a step that is especially important for singletons that might encode strain specific features. To provide such information on-the-fly, the protein sequence observation service (PSOS) was integrated into EDGAR3.0. PSOS allows EDGAR3.0 users to run a predefined set of bioinformatics tools on protein sequences, providing up-to-date information about the functional annotation and classification of a gene. The PSOS analyses can be started with a simple click on the PSOS buttons integrated in the EDGAR3.0 result tables.

### New public database

Based on the new backend described above, it was possible to provide a huge upgrade of the public EDGAR database. As in previous iterations, the genomes of all genera, where more than three finished genomes were available, were downloaded from the NCBI Nucleotide database. Preferably, the RefSeq (24) annotation was used, if a RefSeq annotation was not available, the Genbank (23) annotation was used. If no functional annotation was present for a genome sequence at all, the genome was discarded. This data collection effort resulted in datasets for 523 genera, with 20 032 genomes in total. For all these genera public EDGAR3.0 projects were calculated and made available on the web server. Together with the public type strain projects described in (9), the public database comprises 766 public projects with 24 317 genomes. The project size ranges from the minimum size of three to >1300 genomes (*Escherichia*), while the number of coding sequences per project ranges from 1776 (*Blochmannia*) to >5.5 million. Without the new backend and the use of cloud resources, this update would have been impossible.

## DISCUSSION

The identification of orthologous genes and the calculation of comparative analyses for sets of genomes have become crucial taks in modern genome analysis. Consequently, several other approaches to compute orthologous genes or to provide comparative analyses for sets genomes are available to the scientific community, e.g. GET_HOMOLOGUES (25), Roary (26), OrthoMCL (27), OMA (28) or OrthoFinder (29), or PanX (30). What distinguishes EDGAR from other approaches is its approach to provide precomputed datasets for thousands of public genomes. While the precomputed datasets naturally entail a somewhat lower flexibility than standalone tools, they give EDGAR the ability to quickly provide results even for very large datasets.

As detailed in the introduction, the steadily increasing number of genomes in EDGAR projects has become a limiting factor of the platform. The new backend presented here relies on a scalable technical infrastructure that enables EDGAR3.0 to handle the current amount of publicly available genomic data. The implementation of the BLAST alignment backend as cloud application guarantees the easy scalability of the EDGAR project setup in the future and makes sure that the computation of the initial alignment results will not become the bottleneck of the pipeline.

The modernized storage backend in combination with the fast and memory efficient new API makes the creation of large-scale EDGAR3.0 projects feasible, allowing projects involving hundreds or even thousands of genomes. At the same time, the high performance of the EDGAR3.0 API and the low memory footprint allow more users to utilize the EDGAR3.0 services at the same time without negatively influencing each other. The usage of Go and the application of best coding practices also improved the maintainability of the source code which in turn improves the long-term sustainability of the new implementation.

The computation of orthologs based on the new implementation is deterministic and in the vast majority of cases equivalent to the results of the original implementation. One of the main challenges in orthology estimation is the abundance of paralogous genes. Since EDGAR version 2.0, identical paralogs are filtered during the project setup, but closely related genes within one genome remained a source of bias. To reduce this bias, the best hit estimation has been improved in EDGAR 3.0. In previous version of EDGAR the best hit was determined exclusively based on the score ratio value. Since EDGAR 3.0 the BLAST bit score and percent identity are used as secondary criteria for hits with identical score ratio. This can cause results to slightly diverge from previous implementations, but in a way that newly computed results always show improved ortholog assignment.

The web server was extended with various new features, of which some have been presented in this manuscript. The addition of rapid alternatives to the established ANI/AAI methods broadens the variety of available tools for genome distance calculation. UpSet plots and multi genome circular plots provide new visualizations for complex comparative queries. The integration of the PSOS service allows users to check for up-to-date annotations of genes of interest, which is particularly helpful when analyzing singleton genes, for which information can be drawn from orthologous genes. Together with the set of analysis and visualization features established in EDGAR since the launch of the platform in 2009, EDGAR3.0 provides a comprehensive feature set for comparative genomics and phylogenomics on a new, sustainable technical backend.

While the backend of EDGAR3.0 is capable of handling 1000 genomes and more, for some features of the EDGAR3.0 the number of genomes that can be analyzed had to be limited. For instance the number of genomes for which an AAI matrix can be created had to be limited to 450, as beyond this value the heatmaps get so large that they can no longer be displayed without causing browsers on weaker computers to freeze. The same holds true for the interactive display of result tables. For larger core and pan genome queries, the number of entries in the tables gets too large to be handled by the web server. In such cases, the table data is presented as a downloadable file instead of an interactive table.

The quality of EDGAR analyses is highly dependent on the quality and homogeneity of the annotations used to create the project. For the public projects, EDGAR has to rely on findable and accessible annotations. To ensure maximal comparability, EDGAR3.0 uses the RefSeq annotations, as all genomes in RefSeq are re-annotated using the latest version of the PGAP pipeline (31). For private projects, EDGAR has to rely on the genomes provided by the users. The users are encouraged to perform a uniform and up-to-date re-annotation of the input genomes, e.g. by using the ASA^3^P platform (32).

The methods implemented in EDGAR are available to scientists all over the world as free-to-use service provided by the Bielefeld-Gießen Resource Center for Microbial Bioinformatics (BiGi) as part of the German Network for Bioinformatics Infrastructure (de.NBI). Besides the 24,317 genomes that are available in the 766 projects of the public EDGAR3.0 database section, users can get access to private, password protected projects to analyze unpublished data or arbitrary sets of genomes of their choice. Currently, 966 private projects are hosted on the EDGAR3.0 platform with more than 44 000 genomes for >200 scientific institutions worldwide. Overall, >31 000 genomes have been processed by the EDGAR platform in the year 2020, alone.

## CONCLUSION

The new backend implemented in EDGAR3.0 represents a major step forward for the EDGAR platform. The cloud based alignment tool allows for the timely computation of orthologs for large sets of genomes, and the fast file-based storage solution in combination with the new calculation API allows the platform to handle all queries on considerably larger projects in a fast and efficient way. The scalable implementation of all modules of the new backend ensures that EDGAR3.0 can be easily adjusted to further increasing resource requirements. EDGAR3.0 is a versatile tool used worldwide both in basic taxonomic research and in comparative genome analyses, with currently >30 000 genomes that are analyzed on the platform per year. With the presented updates to the underlying backend, the EDGAR3.0 platform is ready to handle a further increase of available genomic data in the future.

The API implemented for EDGAR3.0 is currently used exclusively by the web server. It would be an evident task, though, to make this API available for external users as well to allow them to compute the comparative genomics results underlying the EDGAR3.0 features via API requests for integration into other software tools or pipelines. The API could also be used for standalone ‘satellite’ applications, e.g. it would be possible to generate an interface that computes phylogenetic trees and genome distance matrices of an uploaded genome in comparison to a public EDGAR project without the need to set up a full featured EDGAR3.0 project.

There are also further updates to the EDGAR3.0 web server planned for the future, with a special focus on usability improvements in large projects. While the backend can now handle huge genome numbers, the process of selecting the genomes of interest can be tedious. Thus, it is planned to introduce genome selection groups in future upgrades, which, after being defined once by the user, can be reused in all EDGAR3.0 features for quick genome selection.

The emphasis for the development of new analysis features will be placed on phylogenomic analyses, e.g. the integration of the state-of-the-art phylogeny approach of the Genome Taxonomy Database (GTDB, (33)) based on 120 marker genes is under development. In general, the new backend will allow a flexible addition of new features to EDGAR3.0, as the architecture of the API allows for the easy implementation of new features as microservices. Based on our recent developments, EDGAR is well on the way to continue to play a key role in comparative genomics in general and phylogenomics in particular.

## ENDNOTES

https://www.denbi.de/cloud.
